# Preterm Infant with Generalized Arterial Calcification of Infancy Who Survived Due to Early Diagnosis and Appropriate Treatment with Bisphosphonates: A Case Report

**DOI:** 10.3390/children11101176

**Published:** 2024-09-27

**Authors:** Masato Tanaka, Akira Kobayashi, Haruhiro Kuwabara, Jun Nirei, Junichi Ozawa, Kentaro Sawano, Nao Shibata, Keisuke Nagasaki, Akihiko Saitoh

**Affiliations:** 1 Department of Pediatrics, Niigata University Medical and Dental Hospital, Niigata 951-8514, Japan; akyura@med.niigata-u.ac.jp (A.K.); hkuwabara@med.niigata-u.ac.jp (H.K.); nirei-j@med.niigata-u.ac.jp (J.N.); jyunichioza.yt2@nuh.niigata-u.ac.jp (J.O.); sawano@med.niigata-u.ac.jp (K.S.); shibata-nao@med.niigata-u.ac.jp (N.S.); nagasaki@med.niigata-u.ac.jp (K.N.); asaitoh@med.niigata-u.ac.jp (A.S.); 2 Department of Pediatrics, Niigata University Graduate School of Medical and Dental Sciences, Niigata 951-8514, Japan

**Keywords:** generalized arterial calcification of infancy, bisphosphonates, arterial calcification, preterm infant

## Abstract

Generalized arterial calcification of infancy (GACI) is a rare disease characterized by arterial calcification. GACI is caused by a mutation in the ENPP1 or ABCC6 genes. GACI causes severe hypertension and heart failure, and approximately 50% of patients die within the first 6 months. In particular, preterm infants with GACI often die due to immature cardiac function. Bisphosphonates are effective in treating GACI; however, no standardized treatment regimen is available. We experienced a case of a preterm infant with GACI born at 30 weeks gestation. Ultrasonography showed high-intensity lesions in the arteries, and computed tomography revealed calcification of the arteries throughout the body, leading to the diagnosis of GACI. We administered intravenous pamidronate, and her cardiac contraction improved. The initial scheduled interval between drug administrations was 2 months. However, the cardiac contraction worsened 1 month after the pamidronate administration. Therefore, we decreased the dosing interval and administered a second course of pamidronate, which improved her cardiac function. We then switched to oral etidronate. To improve the morbidity and mortality rates of preterm infants with GACI, it is important to obtain an early diagnosis of GACI by investigating high-intensity lesions in the arteries and performing early administration of an appropriate type of bisphosphonate.

## 1. Introduction

Generalized arterial calcification in infancy (GACI) is a rare disease with an estimated prevalence of 1 in 200,000 pregnancies [1]. GACI is characterized by calcification of the elastic fibers of the arteries and marked neointimal proliferation, leading to arterial stenosis, resulting in severe hypertension, dyspnea, and heart failure [2]. The diagnosis of GACI is triggered by the presence of high-intensity lesions in the arterial walls on ultrasonography or computed tomography (CT). The diagnosis is confirmed when molecular genetic testing reveals biallelic pathogenic variants in the ectonucleotide pyrophosphatase/phosphodiesterase 1 (ENPP1) gene or the ATP binding cassette C6 (ABCC6) gene. However, GACI is a very rare disease that often goes undiagnosed. In early onset GACI, clinical symptoms such as non-reassuring fetal status, heart failure, hypertension, and hydrops fetalis are observed, and patients often die of myocardial infarction or heart failure within the first 6 months of life [3]. In particular, preterm infants with immature cardiac function are extremely difficult to save.

Of all patients with GACI, approximately 67% have a pathogenic variant in the ENPP1 gene, and 9% have a pathogenic variant in the ABCC6 gene [4]. As a result, patients with GACI have decreased serum inorganic pyrophosphate (PPi) concentrations and have calcium hydroxyapatite crystals deposited in the arteries. Administration of bisphosphonate, which is structurally similar to PPi, is believed to inhibit and eliminate intravascular calcification; however, there is currently no standard treatment regimen [5].

Herein, we describe a very rare case of a preterm infant with GACI born at 30 weeks gestation, who was diagnosed early and provided with appropriate treatment and survived.

## 2. Case Presentation

**Figure 1:**
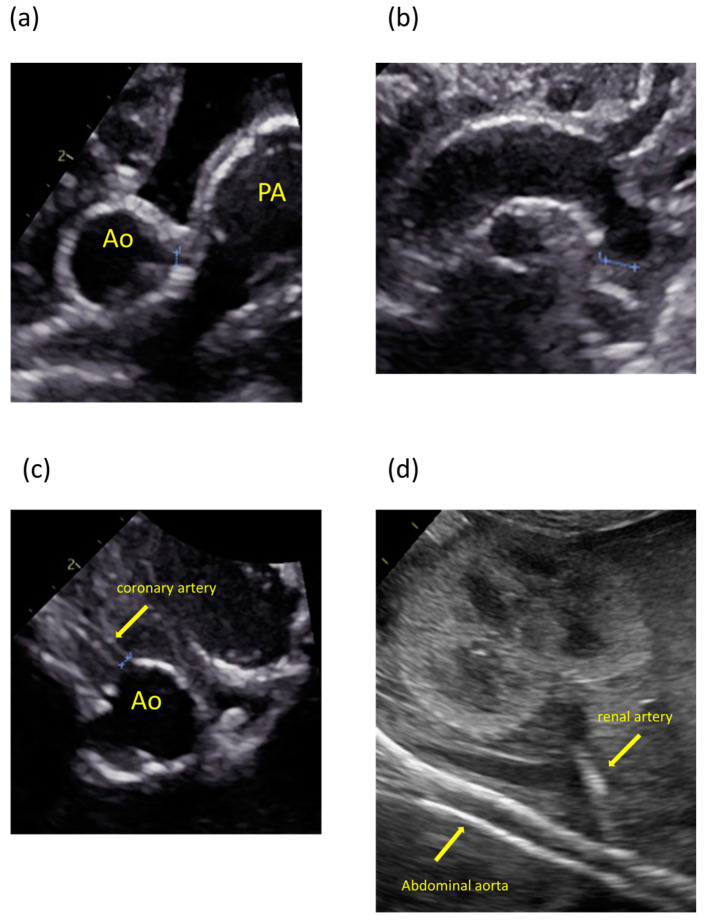
Ultrasonography images showing calcification and high-intensity lesions in the arterial walls. (**a**) Aorta and pulmonary artery. (**b**) Aortic arch. (**c**) Coronary artery. (**d**) Abdominal aorta and renal artery.

The patient was the second child of nonconsanguineous parents without a family history of the disease. The patient’s mother was a 35-year-old pregnant woman, gravida 2 para 1, and had no history of miscarriage. She was transferred to our hospital because fetal ultrasonography showed hydrops fetalis. Her obstetric history was unremarkable, without significant medical or surgical histories. There were no abnormalities at her prenatal checkups until 28 weeks of gestation. Fetal ultrasonography on admission revealed polyhydramnios, hydrops fetalis, and cardiac enlargement. Betamethasone was administered in preparation for premature delivery. A cesarian section was performed at 30 weeks of gestation because of non-reassuring fetal status. The patient was born with a birth weight of 1804 g. The Apgar scores were 1 and 3 at 1 and 5 min, respectively. The patient required resuscitation and tracheal intubation at birth due to respiratory distress syndrome. No dysmorphic features were observed. Postnatal ultrasonography showed enlargement of both ventricles and decreased cardiac contractility, with high-intensity lesions in the walls of the aorta, pulmonary artery, ductus arteriosus, and coronary artery (Figure 1). The blood pressure was markedly high (69/49 mmHg, upper 95th percentile).

We started treatment with continuous intravenous dobutamine and intravenous furosemide, and the cardiac contractions improved. However, her blood pressure increased from 4 days of age (systolic pressure was over 80 mmHg), and her cardiac contractions decreased accordingly. Considering afterload mismatch, carperitide was administered at 5 days of age. The initial dose was 0.05 µg/kg/min and was increased to 0.1 µg/kg/min, but her blood pressure did not decrease. From 7 days of age, nitroglycerin was administered at a starting dose of 0.5 µg/kg/min and was increased to 2 μg/kg/min, but no effect was seen as the cardiac contractions did not improve.

**Figure 2:**
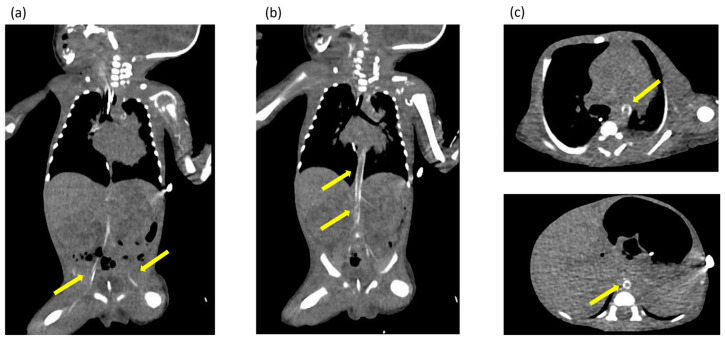
(**a**–**c**) Computed tomography images obtained at 17 days of age showing extensive calcification of the arterial walls throughout the body. The yellow arrows indicate areas of calcification identified in the arterial wall throughout the body.

Ultrasonography of the chest revealed enhanced high-intensity lesions of the arterial walls. The high-intensity lesion persisted and gradually intensified. We suspected calcification of the blood vessels and performed CT at 17 days of age, which revealed calcification of the arterial walls in her whole body (Figure 2). No extravascular calcification was noted, and medullary nephrocalcinosis was not seen. We diagnosed the patient with GACI due to severe arterial calcification.

**Figure 3:**
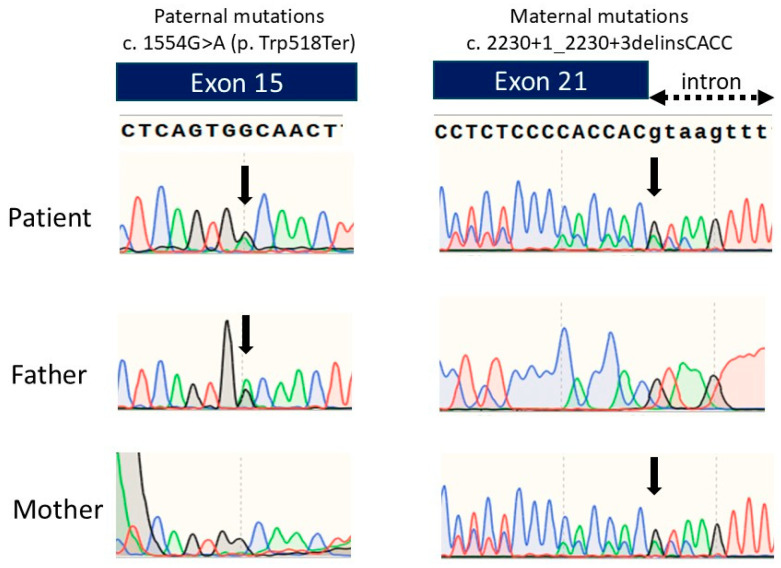
Chromatograms of the DNA sequence of exon 15 and exon 21 of the *ENPP1* gene in the patient and the parents. Green line represents Adenine, Blue line represents Cytosine, Black line represents Guanine, and Red line represents Thymine.

We performed an *ENPP1* gene analysis and detected two heterozygous variants: c.1554G>A(p.Trp518Ter) and c.2230+1_2230+3delinsCACC. An *ENPP1* gene analysis of her parents detected the heterozygous pathogenic variant c.2230+1_2230+3delinsCACC in the mother’s *ENPP1* gene and c.1554G>A(p.Trp518Ter) in the father’s *ENPP1* gene (Figure 3). The mother’s pathogenic variant was previously reported, while the father’s variant is novel. The mother’s pathogenic variant is speculated to produce abnormally sized ENPP1 proteins due to splice error, leading to loss of function.

The patient had a low serum PPi concentration of 0.11 µM (normal range 2.36–4.44 µM [6]). We obtained permission to administer bisphosphonate to the patient after an ethical review by Niigata University Hospital. We explained the treatment to the patient’s parents and obtained their consent to begin administering bisphosphonate. Intravenous administration of pamidronate (three infusions of 0.25, 0.50, and 0.50 mg/kg on days 18, 19, and 20 after birth, respectively) was performed because the patient’s general condition was poor and enteral nutrition had not been established. After treatment with pamidronate, the left ventricular ejection fraction rate improved from 19% to 51%, but we initiated treatment with nifedipine because the hypertension persisted. We regularly observed the arterial walls by ultrasonography and revealed no significant changes in the high-intensity lesions of the coronary, pulmonary, aorta, and renal arterial walls.

**Figure 4:**
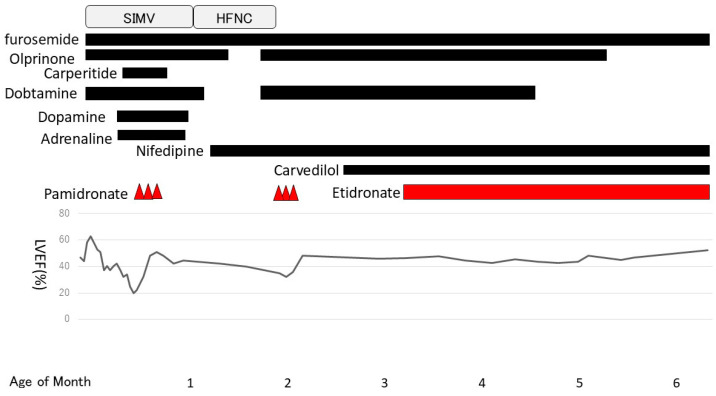
Clinical course. Pamidronate was started at 18 days of age. The second course was started at 60 days of age and was switched to etidronate at 98 days of age. SIMV: Synchronized Intermittent Mandatory Ventilation. HFNC: High Flow Nasal Cannula.

One month after pamidronate administration, her cardiac function worsened again. Her left ventricular ejection fraction had been in the 40% range until then but gradually decreased, and enlargement of the left ventricle and left atrium became prominent. Mitral regurgitation, which had previously been mild, became severe, and increased afterload was observed. We resumed dobutamine and olprinone at 54 days of age, but there was no improvement in cardiac contractions. Normally, the treatment cycle would have been every 2 months; however, we decided to start the second course early to address the worsened cardiac function. The second course was started 3 weeks ahead of the schedule at 60 days of age (0.5 mg/kg for 3 days) and was changed to oral disodium etidronate (20 mg/kg/day) at 98 days of age. After the second course of pamidronate in addition to dobutamine and olprinone, her cardiac contraction gradually improved. The left ventricular and atrial enlargement tended to improve slightly, and mitral regurgitation also became less severe. To treat heart failure, we introduced carvedilol (0.01 mg/kg/day) at 72 days of age and continued dobutamine until 128 days of age (Figure 4).

**Figure 5:**
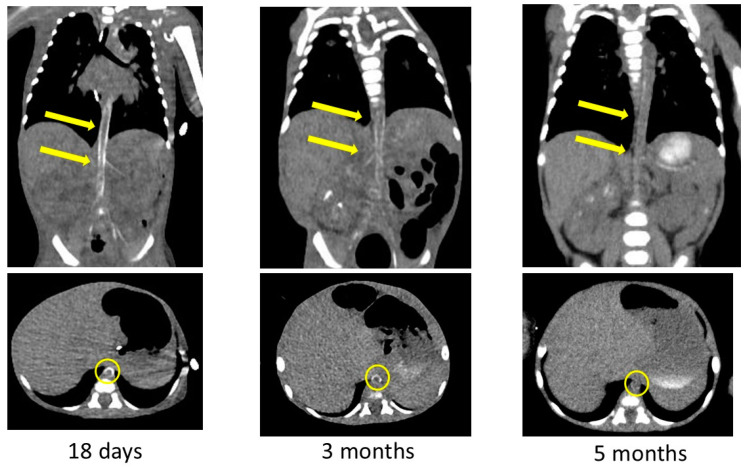
Contrast-enhanced computed tomography images showing decreased calcification of the arteries after bisphosphonate treatment. Yellow arrows and circles indicate improvement in arterial wall calcification in the same area.

The arterial calcification was decreased on CT images obtained at 3 and 5 months after birth (Figure 5). We had observed high-intensity lesions of the arterial wall by ultrasonography, which began to lighten around 3 months of age and were virtually unnoticeable at 5 months of age, as well as CT images. She was discharged to home 6 months after birth on diuretics, aspirin, antihypertensive medications, and etidronate medications. We performed a hearing test with automated auditory brainstem response, and no hearing loss was found. She was examined by a retina specialist, and her retinal findings were normal.

## 3. Discussion

We experienced a case in which a preterm infant with GACI born at 30 weeks of gestation survived due to the early diagnosis and appropriate treatment with bisphosphonates. GACI has a poor prognosis because it causes early calcification of arterial vessels, which rapidly leads to hypertension, heart failure, and multiple organ failure. About 55% of patients with GACI die within the first 6 months of life [7]. One study reported that 12% of infants with GACI die in utero, 24% die at less than 1 day of age, and 25% die at less than 1 month of age [7]. In particular, few preterm infants with GACI who have low cardiac function and capacity survive. We speculated that there were two reasons that our preterm patient with GACI survived. First, we diagnosed the patient with GACI early after birth and administered the first dose of bisphosphonate before the disease condition worsened. Second, we were able to adjust the dosing interval of bisphosphonate in response to her cardiac condition.

GACI is a rare disease that is estimated to occur in 1 in 200,000 pregnancies [1]. Because this disease is not well recognized by pediatricians, obstetricians, and neonatologists, it is difficult to diagnose GACI appropriately. In fact, some cases are diagnosed by autopsy, and some cases are undiagnosed [8,9,10]. Our suspicion of GACI was triggered by the presence of clinical symptoms such as hydrops, hypertension, and heart failure, in addition to the appearance of high-intensity lesions in the arterial walls on ultrasonography.

The arteries most frequently noted to be calcified in GACI are the aorta, hepatic artery, coronary artery, and renal artery [11]. Therefore, clinicians should suspect GACI when ultrasonography shows high-intensity lesions in these arteries. The present case also showed characteristic findings on ultrasonography. In early onset GACI, similar findings are detected in utero [12]. A review of the present patient’s medical records revealed that there was a high-intensity lesion of the aorta detected on fetal ultrasound. It is important to suspect GACI when a patient presents with unexplained hydrops, hypertension, heart failure, and persistent or enhanced high-intensity lesions of the arterial walls on ultrasound. If GACI can be diagnosed prenatally, appropriate counseling can be provided to the parents [13,14]. This would also lead to proper management of the fetus during pregnancy and early initiation of treatment after birth.

We were able to administer bisphosphonate as early as 18 days of age. The afterload does not reduce in GACI unless the calcification of the vessel wall is improved. Therefore, the usual treatment of heart failure with catecholamines, vasodilators, and diuretics has limited efficacy. Bisphosphonate is the most widely used drug to treat GACI. Bisphosphonate is similar to PPi in molecular structure, and it improves the calcification of vessels when administered to patients with GACI with reduced PPi concentrations [5]. The survival rate of patients with GACI who are administered bisphosphonate is 65%, which is much higher than the survival rate of 31% for patients with GACI who are not administered bisphosphonate [15]. As more than half of patients with GACI die within the first month of life, early administration of bisphosphonate is important.

On the other hand, some have been critical of the efficacy of bisphosphonate. Ferreria CR et al. reported bisphosphonate had a significant effect only when administered within 7 days of birth and no significant effect when administered later [16]. Conversely, this suggests the importance of early diagnosis. In the present case, the administration of bisphosphonate was initiated at 18 days of age. Indeed, we cannot rule out the possibility that the improvement of cardiac function in this case was caused by a combined effect of bisphosphonates and other drugs. After 7 days of age, the administration of bisphosphonates alone may not be sufficient. However, it is also true that improvement in cardiac function was observed after the administration of bisphosphonates. Therefore, bisphosphonates can have a certain effect in reducing cardiac burden. GACI is a disease with a poor prognosis, and its treatment has not yet been established. In addition, newborns, especially preterm infants, have immature cardiac function. Given these circumstances, the administration of bisphosphonate should be considered for newborns and preterm infants with GACI, even if they have a small effect in reducing cardiac burden.

In the present case, the bisphosphonate dosing interval was shortened because the patient’s heart failure had worsened. The treatment regimen of bisphosphonate for GACI has not yet been standardized. The commonly used treatment protocols are oral etidronate (20 mg/kg/day), intravenous pamidronate (0.25 mg/kg on day 1 and 0.5 mg/kg on days 2 and 3 for the first course, and 0.5 mg/kg for 3 days as the second course, with courses administered every 2 months), and oral risedronate (1 mg/kg/week) [2]. The non-nitrogen-containing bisphosphonate etidronate is a first-generation bisphosphonate and is the most used because of its close structure to PPi. Etidronate, which has a structure similar to PPi, inhibits osteoclast function and has the effect of suppressing calcification [17]. In contrast, nitrogen-containing bisphosphonates, such as pamidronate and risedronate, inhibit the action of enzymes in the mevalonate pathway, thereby suppressing bone resorption by osteoclasts [18]. Etidronate has been shown to inhibit phosphate-induced calcium deposition more potently than nitrogen-containing bisphosphonates. Therefore, for the purpose of improving calcification, etidronate appears to be a better option than other bisphosphonates [19]. However, there are no reports comparing the therapeutic effects of nitrogen-containing bisphosphonates with those of non-nitrogen-containing bisphosphonates in the treatment of GACI. In this case, there was no recurrence of calcification symptoms after switching to etidronate, and etidronate might have contributed to the improvement in calcification.

There have been some reported cases in which treatment was started with pamidronate and switched to oral etidronate once the patient’s general condition stabilized [20,21]. The current patient had poor general condition, and enteral nutrition had not been established, so the initial treatment was started with pamidronate, which improved her heart failure. Although the pamidronate was supposed to be administered every 2 months, the cardiac function of our patient worsened again 1 month after the initial pamidronate administration. Hence, the second course was started 3 weeks earlier than scheduled, considering the possibility that the calcification had worsened. The cardiac function of the patient subsequently improved, and she was then switched to oral etidronate. If we had waited until the recommended time to administer the second course of pamidronate, her cardiac function might have deteriorated further. When using pamidronate, consideration should be given to shortening the dosing interval based on the patient’s cardiac function. However, in the present case, enteral nutrition was established after the first course of pamidronate. In hindsight, we should have switched to oral etidronate at that time point.

A clinical trial of enzyme replacement therapy in ENPP1 deficiency is currently ongoing. The outcomes of this trial may lead to significant improvements in future treatment strategies [22,23].

This case report’s limitation is that we could not reach a diagnosis of GACI immediately when we checked the high-intensity lesions in the arterial wall on ultrasonography. Initially, we had not recognized if the high-intensity lesions in the arterial wall on ultrasonography were of pathological significance. However, the persistence and enhancement of the high-intensity lesions in the arterial wall allowed us to suspect and diagnose GACI. This ultrasonography finding, which is characteristic of GACI, needs to be widely recognized. The other is that we did not evaluate the calcification status with CT before starting the second course of pamidronate. We assumed that arterial calcification had worsened because of decreased cardiac function and increased afterload. However, we should have performed CT imaging at that time to evaluate the progression of calcification.

## 4. Conclusions

We experienced a case in which a preterm infant with GACI survived because of an early diagnosis and appropriate treatment. GACI should be suspected when high-intensity lesions in the arterial wall are observed on ultrasound or CT. Bisphosphonates may have limited efficacy for GACI, but their use should be considered in neonates and preterm infants with immature cardiac function. When used, clinicians should choose the type of bisphosphonate depending on the patient’s situation. Once enteral nutrition is established, clinicians may consider administering oral etidronate. Appropriate treatment regimens for GACI are needed.

## Data Availability

All data are contained within the article.
